# Asylum Worthies

**Published:** 1898-02-19

**Authors:** 


### The Institutional Workshop.

### ASYLUM WORTHIES.

### MR. RICHARD GREENE, F.R.C.P.Edin.

Mr. Richard Greene, who has just retired from the medical superintendentship of the County Asylum, Northampton, has had thirty years' experience in lunacy. During the whole of that period he has had an unbroken record of success, which has redounded to his credit and elevated him to a leading position among the lunacy experts of the day. It is interesting to note in this connection that from seven to ten years' service as assistant medical officer of an asylum represents the training which is held to be adequate to qualify for the post of medical superintendent in this country. If a man remains an assistant medical officer for a much longer period than ten years, every year his chances of being appointed to the higher office diminish rather than increase. The reason for this is no doubt to be found in the fact that if an assistant medical officer does not succeed in making his personal claims recognised within the first ten years the prospects of his promotion are not great.

Mr. Richard Greene took the double qualification at the Royal College of Physicians and Surgeons, Edinburgh, in 1868. He passed the L.S.A. examination in 1876, became a member in 1885, and a Fellow of the Royal College of Physicians, Edinburgh, in 1888. During his student career he took first-class honours in surgery, and was a silver medallist in chemistry. He was the author of a number of papers dealing with hospitals for the insane, clinical instruction in asylums, asylum hospitals with plans, care and cure of the insane, the hygiene of asylums, the construction and arrangement of asylums, and has been successful in more than one competition for designs for new hospitals for the insane, including those for the Derby Borough Asylum, for the Blackburn, and the Isle of Wight asylums. This knowledge of construction has proved of great value to the county of Northampton. For nearly twenty years, although many additions to the asylum and asylum buildings have been made, and notably a fever and infectious diseases hospital, the children's block costing about £3,500, and about 15 residences for the staff have been built, not one penny has ever been expended by the committee on architects' fees, Mr. Greene having prepared the designs and superintended the buildings entirely by himself. The savings thus effected have been considerable, but they are nothing as compared with the savings which have been effected in other directions. All the foregoing buildings have been provided, and several purchases of land have been made, without the county being called upon to pay any sum towards this expenditure. In July, 1878, when Mr. Richard Greene assumed office at Berrywood, the total number of patients was 523, and the cost of maintenance per head per week was 10s. 6d. The patients now amount to an average of about 900, and by gradually decreasing stages the cost of maintenance of patients per head per week has been reduced from 10s. 6d. to 7s. 6d. The saving thus effected in the maintenance charge is equal to a sum of nearly £5,500 per annum. In addition to this, owing to the excellence of the management, the Committee of Visitors have been enabled to return to the county a sum of money in aid of the rates at the close of 1896 approaching £10,000, in addition to a reserve fund standing to the credit of the building and repairs account of nearly £5,000. Such results as these speak for themselves. They prove to demonstration that where a committee of visitors has the ability to select a medical superintendent of knowledge and judgment, the county they represent is able to reduce the charge on the rates for the maintenance of its insane to a minimum, whilst the efficiency of the administration is kept up to the highest point of perfection.

There are, of course, a great number of people who object to a pension being granted to anyone but themselves. It is rather surprising, therefore, despite the eminent services rendered to the county of Northampton by Mr. Richard Greene during the last twenty years, and at the same time highly creditable to the constituents of the Northampton County Council and their representatives, that a handsome pension should have been granted to Mr. Greene at a full meeting with practical unanimity. We have no sympathy with the men, who are usually very rich and well-to-do, who make it their business to belittle the services rendered by high officials upon whose fidelity and devotion the successful management and the high reputation of our public establishments must mainly depend. Still, the action of the Northampton County Council is noteworthy because it proves that public bodies can and do recognise the importance to the commonwealth of such services, and that they are prepared upon occasion to liberally recognise the devotion of high officials who deserve well of their age and generation.

Anyone who has inspected the Northampton County Lunatic Asylum, and all who knew it before the days of Mr. Greene's superintendentship, will cordially acknowledge that his reign has had a most material effect for good upon the efficiency and character of the whole institution. The Northampton County Lunatic Asylum to-day is not only one of the best administered, but one of the best planned, most complete, and attractive hospitals for the insane in this country. No modern improvement either in construction or in system has been overlooked, and Berrywood Asylum has the distinction of being practically the only institution of the kind in the United Kingdom where an adequate system for training the attendants has been organised, to the great advantage of the patients and the community at large. Berrywood was one of the first English asylums to adopt the open door system, which has proved entirely successful, and has justified the wisdom and courage of the men by whose instrumentality it has been maintained there for so many years. Mr. Richard Greene has been very successful with his sewage farm. He is an expert landscape gardener, and the grounds, and indeed the whole of the Berrywood estate, will remain as a practical monument to his skill in this kind of work. Personally, Mr. Greene possesses many excellent gifts. He has read much, and has proved himself to be a centre of social influence within the county, has raised the standard of asylum work by his example and personal traits, and has set an example in devotion to the duties of his office which has won for him the respect and esteem not only of his committee but of all who take an intelligent interest in the welfare of the mentally afflicted. He has studied interior decoration as an art, has acquired a knowledge of engravings and pictures which is as rare as it is valuable, and a visit to his residence is an education in design owing to the wonderful collection of the best types of Chippendale and other furniture it contains. The staunchest of friends, the brightest of companions, a devoted father and husband, Dr. Greene may be truthfully said to combine in his own person most of the attributes which constitute the best possession of an English gentleman. He is very fond of travel, and during his life has visited nearly all the countries of Europe, and is probably better acquainted with the various systems of asylum administration than almost any man now living. We have no doubt, and it is not surprising that such should be the case, that he will be greatly missed at Berrywood, and the heartfelt regret of himself at his enforced retirement through failing health will be shared by everyone who has had the privilege of serving under him, as well as by everyone who takes an interest in the progress and development of the county of Northampton.

